# Determinants of Weight Gain during the First Two Years of Life—The GECKO Drenthe Birth Cohort

**DOI:** 10.1371/journal.pone.0133326

**Published:** 2015-07-20

**Authors:** Leanne K. Küpers, Carianne L’Abée, Gianni Bocca, Ronald P. Stolk, Pieter J. J. Sauer, Eva Corpeleijn

**Affiliations:** 1 Department of Epidemiology, University Medical Center Groningen, University of Groningen, Groningen, the Netherlands; 2 Department of Pediatrics, Beatrix Children’s Hospital, University Medical Center Groningen, University of Groningen, Groningen, the Netherlands; Hamamatsu University School of Medicine, JAPAN

## Abstract

**Objectives:**

To explain weight gain patterns in the first two years of life, we compared the predictive values of potential risk factors individually and within four different domains: prenatal, nutrition, lifestyle and socioeconomic factors.

**Methods:**

In a Dutch population-based birth cohort, length and weight were measured in 2475 infants at 1, 6, 12 and 24 months. Factors that might influence weight gain (e.g. birth weight, parental BMI, breastfeeding, hours of sleep and maternal education) were retrieved from health care files and parental questionnaires. Factors were compared with linear regression to best explain differences in weight gain, defined as changes in Z-score of weight-for-age and weight-for-length over 1–6, 6–12 and 12–24 months. In a two-step approach, factors were first studied individually for their association with growth velocity, followed by a comparison of the explained variance of the four domains.

**Results:**

Birth weight and type of feeding were most importantly related to weight gain in the first six months. Breastfeeding versus formula feeding showed distinct growth patterns in the first six months, but not thereafter. From six months onwards, the ability to explain differences in weight gain decreased substantially (from R^2^_total_ = 38.7% to R^2^_total_<7%).

**Conclusion:**

Birth weight and breast feeding were most important to explain early weight gain, especially in the first six months of life. After the first six months of life other yet undetermined factors start to play a role.

## Introduction

In 2009 in the Netherlands, 12.8% of the boys and 14.8% of the girls (2–21 years) were overweight and 1.8% of the boys and 2.2% of the girls were obese [1]. Childhood overweight and obesity are associated with many co-morbidities [2,3]. Overweight children frequently become overweight adults [4,5].

Prenatal and postnatal growth in the first half year of life are known to be essential for both infant growth (0–2 years) and later development[4,6,7]. Studies have indicated that a rapid weight gain in infancy is related to an increased risk of obesity during childhood and later in life [7,8]. It is not clear from these studies which period in early life might be most important for the development of overweight, and growth in the first 4–6 months of life has not extensively been studied yet [4,8]. Early detection of risk factors for sustained excessive weight gain is essential, as there is increasing evidence that prevention and treatment of obesity at a very early age is most effective [9,10].

Studies have shown that formula fed infants have a higher chance to become obese compared to breast fed infants [11]. Whether this is related to a difference in growth pattern in the first six month of life is unknown. Although studies evaluated the difference between breast and formula feeding on the development of overweight, other factors like prenatal factors, sleep duration, screen time and socioeconomic factors have rarely been tested simultaneously in one model [12]. In order to prevent the development of obesity in later life, it is important to identify those factors that are most important for explaining infant growth, relative to the other potential risk factors. Additionally, it is unclear exactly in which period in the first years of life (e.g. 0–1 month, 1–6 months, 6–12 or 12–24 months) most of the rapid infant growth occurs, since many studies evaluated rather long growth intervals [8,13–15]. The GECKO Drenthe birth cohort is an excellent setting to study early infant growth in detail, because of its many repeated anthropometric measurements and questionnaires.

In the GECKO Drenthe birth cohort, we collected data regarding maternal pre-pregnancy weight, weight gain, diseases and lifestyle during pregnancy, birth weight and the postnatal weight gain and lifestyle, and we grouped these into four domains comparable to the well-known health fields conceptual framework by Lalonde [16]. From these data, we aimed to analyze which factors from the various important domains, i.e. prenatal, nutrition, lifestyle and socioeconomic domains were most importantly associated with gain in weight during the first two years of life. Special attention was paid to the variation in growth velocity over different time frames within the first two years of life.

## Methods

The GECKO Drenthe birth cohort has been designed to study the determinants of overweight in early life. All parents from children born from April 2006 to April 2007 and living in Drenthe, a northern province of the Netherlands, were invited to participate. Mothers were invited to participate in the third trimester of their pregnancy. Detailed information has been published elsewhere [17]. During childhood, multiple anthropometric measurements were performed by trained staff at Well Baby Clinics. Body weight was measured using an electronic scale with digital reading to the nearest 0.01 kg. Length was assessed using an infantometer to the nearest 0.1 cm. Measurements were performed in supine position until the age of 2 years. Birth weight was obtained from midwives’ records. For the current study, growth data at 0, 1, 6, 12 and 24 months were used. This study was approved by the Medical Ethics Committee of the University Medical Center Groningen and performed in accordance with the Declaration of Helsinki and all parents gave written informed consent.

We selected several prenatal, nutritional, lifestyle and socioeconomic factors that have previously been linked to infant growth and/or childhood overweight [13,15,18,19]. These were all self-reported by the parents, except for gestational age and birth weight, which were measured by midwives. All variables and their categorization are shown in Table 1. Maternal diabetes (pre-existing and gestational) and hypertension were defined as ever diagnosed by a medical doctor in the past or during this pregnancy. Smoking during pregnancy was defined as any maternal smoking during pregnancy versus neither maternal nor paternal smoking during pregnancy. Early complementary feeding was defined as fruit/vegetable, porridge, other or none at four months (“other” included dessert, cookies or fruit juice). Family screen time was defined as hours of television viewing per day by any family member in presence of the child at the age of 6 months. Hours being able to move unrestrictedly was defined per day at the age of 9 months. For movement data we did not test the association with weight change for the first six months, because this potential risk factor was measured after that period. This also held for television watching. Following the conceptual health framework by Lalonde [16], we used the methodological approach of grouping all these potential risk factors into four domains: prenatal factors (Lalonde’s human biology field), infant nutrition (Lalonde’s lifestyle field), infant lifestyle (Lalonde’s lifestyle field) and family socioeconomic factors (Lalonde’s environment field). We chose to separate nutrition and other lifestyle factors, because nutrition is such a dominant energy-balance related factor. The classification of the individual factors in the domains can be found in Table 1. These analyses were performed in a two-step approach: first we were interested in associations of growth with individual factors within the domains, followed by a comparison of the explained variance between these four domains.

**Table 1:** Descriptive data of study population (n = 2475).

*Prenatal factors*	
Gestational age (weeks)	39.6 ± 0.03
Birth weight (kg)	3.59 ± 0.01
Female gender	1239 (50.1%)
Maternal pre-pregnancy BMI	23.9 ± 5.4 IQR
Paternal BMI	25.2 ± 4.2 IQR
Gestational weight gain mother (kg)	13.8 ± 0.13
Smoking during pregnancy	356 (14.4%)
Maternal age at date of birth (years)	33.4 ± 0.10
Maternal diabetes (pre-existing and gestational)	87 (3.5%)
Dutch ethnicity	2294 (92.7%)
Maternal hypertension	266 (10.7%)
*Nutrition*	
Type of feeding at 3 months	
Exclusive (including pumped) breastfeeding	827 (33.4%)
Combined breastfeeding and bottle feeding	296 (12.0%)
Exclusive bottle feeding	1352 (54.6%)
Complementary feeding at 4 months	
None	1540 (62.2%)
Fruit and/or vegetables	525 (21.2%)
Porridge (possibly incl. fruit, vegetables or other)	363 (14.7%)
Other	47 (1.9%)
*Infant lifestyle*	
Sleep at 4 months (hours)	14.8 ± 0.06
Family screen time at 6 months (hours)	1.8 ± 0.04
Possibility of unrestricted moving at 9 months (hours)	4.5 ± 0.05
*Socioeconomic factors*	
Household income	
< €850 per month	31 (1.2%)
€850–1150 per month	86 (3.5%)
€1151–3050 per month	1637 (66.1%)
€3051–3500 per month	415 (16.8%)
≥ €3501	306 (12.4%)
Maternal educational level	
Low/middle	1558 (62.9%)
High	917 (37.1%)
Family structure	
Two-parents family	2401 (97.0%)
One-parent family / co-parenthood / other	74 (3.0%)
Multiparity	1509 (61.0%)
Childcare by family or friends at 3 months	1012 (40.9%)
Mother working at 3 months after delivery	939 (37.9%)

Results are shown as mean±SE or n (%). Maternal and paternal BMI are shown as median±IQR.

For all participants, age- and gender-specific Z-scores of weight-for-age and weight-for-length were calculated in the Growth Analyser software, version 3.5 [20]. These Z-scores for individual observations were calculated based on Dutch growth references from 1997 [21]. We calculated Z-scores at four ages: 1, 6, 12 and 24 months, the median exact ages were 5, 26, 50 and 110 weeks, respectively. Weight change was defined as change in weight-for-age and weight-for-length Z-score in periods 1–6 months, 6–12 months and 12–24 months. A Z-score change of 0 indicated weight change according to their expected growth curve. A positive Z-score change (incline) indicated relative fast weight change, in other words a higher weight gain than expected from the growth curve, and a negative Z-score change (decline) indicated relatively slow weight change, in other words, a lower weight gain than expected. In general the change in Z-score indicated how much infants deviate from their expected growth rate. Furthermore, we calculated age- and gender adjusted weight-for-age and weight-for-length Z-scores at birth based on the birth weight data of the Dutch PIAMA birth cohort with ~4000 participants born in 1996–1997 [22], since birth weight data were not available in the Growth Analyser software. This method was chosen to keep Z-scores at birth comparable to the other Z-scores, which were calculated based on the Dutch Growth Study from 1997 [21]. We have also calculated cohort-specific Z-scores at all time points based on our GECKO data, which showed similar growth patterns (data not shown). We used the Z-scores based on the 1997 reference dataset, because at that time the obesity trend was not (clearly) present yet.

### Statistical analysis

Missing data on covariates were imputed (max. 33% missing on hours of television and anthropometry at 24 months and min. 0.5% missing on maternal age) using multiple imputation, for which we computed 5 imputation datasets. For this imputation we used all covariates and length and weight at the four time points as predictors.

First, we tested correlations between potential risk factors within each domain. If two variables were highly correlated (r>0.60) we deleted the one with the lowest correlation with weight gain. Paternal age was correlated with maternal age (r = 0.68, p<0.001) and placental weight was correlated with birth weight (r = 0.63, p<0.001), thus we deleted paternal age and placental weight from the analyses. Then, for the final linear regression model we analysed the variables from all domains together to prospectively predict weight gain between 1–6, 6–12 and 12–24 months. Since we used multiple imputation, we showed the pooled betas and confidence intervals and the mean R-squares of the five imputed datasets for the explained variance per domain. We used p<0.05 for significance threshold. We constructed hypothesis-generating graphs to support the interpretation of the results e.g. to gain insight in the complex influence of nutrition on weight gain in the first year of life. These graphs show crude, unadjusted Z-scores for each time point, stratified by categories of the potential risk factor.

Statistical analyses were performed in SPSS Statistics version 20.0 (SPSS, Chicago IL, USA) and graphs were constructed using Graphpad Prism 5.04 (GraphPad Software Inc., USA).

## Results

During the cohort recruitment period a total of 4778 infants were born [17]. Parents of 3631 (76.0%) children were informed about the cohort. Parents of 2997 (62.7%) children intended to start in this cohort and parents of 2874 (62%) children have ever actively participated. For the current analysis twins, preterm born infants (<37 weeks), those who stopped participation or those with missing data on anthropometrics at all four studied ages were excluded (n = 346), resulting in 2528 participants. Moreover, we excluded 53 children for missing ≥50% of all covariates, resulting in 2475 children for analysis.

The descriptive data of the participants are shown in Table 1. Half of the children were boys, the mean birth weight was 3590 grams, most children had parents from Dutch descent and one third was exclusively breastfed at three months of age. Birth weight, gestational age and maternal age at birth were comparable to the Dutch national average (3.43 kg, 39–41 weeks and 31 years, respectively) in 2011–2014 [23–25]. Table 2 shows growth in absolute measures for weight and exact age at the time of measurement from birth until 24 months. Next, body weight growth was calculated as the change in age and gender adjusted Z-score over 1–6, 6–12 and 12–24 months. The variation in body weight growth could best be explained for weight-for-age in the period of 1–6 months, with a total explained variance (R^2^) of 38.7% (Table 3). When comparing domains we observed the highest explained variance for the prenatal domain (R^2^ = 1.8–26.6% for weight-for-age Z-score and R^2^ = 2.6–3.8% for weight-for-length Z-score between 1–6 months), followed by the nutrition, the socioeconomic and the lifestyle domain, with the exception of nutrition at 12–24 months for weight-for-age Z-score, which became more meaningful than the prenatal domain (Table 3). To answer the question which individual factors were involved, the results of the multiple regression models for change in weight-for-age and weight-for-length Z-score are presented in Tables 4 and 5, respectively, and findings are explained below per time period.

**Table 2:** Anthropometric data of the participating infants.

	Birth	1 month	6 months	12 months	24 months
Boys weight (kg)	3.66 ± 0.014	4.67 ± 0.018	8.03 ± 0.025	10.17 ± 0.030	13.49 ± 0.054
Girls weight (kg)	3.52 ± 0.014	4.40 ± 0.016	7.50 ± 0.024	9.49 ± 0.029	12.82 ± 0.046
Total weight (kg)	3.59 ± 0.010	4.54 ± 0.013	7.76 ± 0.018	9.83 ± 0.022	13.16 ± 0.033
Boys length (cm)	51.2 ± 0.085	55.3 ± 0.064	68.4 ± 0.069	76.4 ± 0.074	90.2 ± 0.107
Girls length (cm)	50.5 ± 0.088	54.3 ± 0.063	66.6 ± 0.070	74.7 ± 0.076	89.0 ± 0.105
Total length (cm)	50.9 ± 0.062	54.8 ± 0.047	67.5 ± 0.052	75.6 ± 0.055	89.6 ± 0.077
Boys age (wk)	-	4.96 ± 0.03	25.5 ± 0.08	49.7 ± 0.07	110.6 ±0.24
Girls age (wk)	-	4.98 ± 0.03	25.6 ± 0.07	49.6 ± 0.07	110.3 ± 0.24
Total age (wk)	-	4.97 ± 0.02	25.24 ± 0.05	49.63 ± 0.05	110.4 ± 0.17

Results are shown as mean ± SE.

**Table 3:** Explained variances for body weight growth, per domain and per period.

	Prenatal	Nutrition	Lifestyle	Socioeconomic	Total model
*Change in weight-for-age Z-score*					
1–6 months	.266	.107	.006	.008	.387
6–12 months	.018	.008	.006	.013	.045
12–24 months	.022	.031	.005	.010	.069
*Change in weight-for-length Z-score*					
1–6 months	.037	.028	.000	.011	.075
6–12 months	.038	.008	.004	.013	.063
12–24 months	.026	.013	.008	.011	.058

R-squared per domain and for total models as presented in Tables 4 and 5.

**Table 4:** Multivariate regression: factors associated with weight-for-age Z-scores change*.

	1–6 months		6–12 months		12–24 months	
*Prenatal factors*	β	95% CI	β	95% CI	β	95% CI
Gestational age (weeks)	**-0.089**	**-0.120; -0.058**	-0.010	-0.031; 0.011	-0.006	-0.016; 0.004
Birth weight (kg)	**-0.872**	**-0.957; -0.787**	**-0.074**	**-0.129; -0.019**	0.016	-0.010; 0.043
Female gender	**-0.157**	**-0.228; -0.087**	-0.050	-0.104; 0.004	0.002	-0.029; 0.033
Paternal BMI	0.007	-0.004; 0.017	**-0.002**	**-0.007; 0.004**	0.001	-0.002; 0.003
Maternal pre-pregnancy BMI	0.003	-0.006; 0.013	0.004	-0.002; 0.010	0.005	0.000; 0.011
Gestational weight gain mother (kg)	0.002	-0.005; 0.010	0.063	-0.012; 0.139	0.002	-0.037; 0.041
Smoking during pregnancy	0.029	-0.103; 0.162	0.010	0.002; 0.018	0.001	-0.002; 0.004
Maternal age at date of birth	0.001	-0.011; 0.013	0.006	-0.001; 0.013	0.001	-0.002; 0.005
Maternal diabetes	-0.162	-0.377; 0.053	-0.073	-0.203; 0.056	0.021	-0.046; 0.089
Maternal hypertension	-0.018	-0.146; 0.110	-0.005	-0.086; 0.076	-0.017	-0.092; 0.059
Dutch ethnicity	0.056	-0.090; 0.203	-0.083	-0.202; 0.037	-0.026	-0.089; 0.037
*Nutrition*						
Type of feeding at 3 months						
Combined breast and bottle feeding (vs. exclusive breastfeeding)	**0.362**	**0.227; 0.497**	0.073	-0.047; 0.193	**-0.046**	**-0.086; -0.005**
Exclusive bottle feeding (vs. exclusive breastfeeding)	**0.759**	**0.669; 0.849**	-0.047	-0.120; 0.026	**-0.081**	**-0.108; -0.055**
Complementary feeding at 4 months						
Fruit and/or vegetables (vs. no compl. feeding)	-0.059	-0.163; 0.045	-0.030	-0.098; 0.038	-0.002	-0.031; 0.027
Porridge, possibly with fruit/veg/other (vs. no compl. feeding)	0.090	-0.038; 0.217	-0.005	-0.094; 0.085	-0.023	-0.050; 0.005
Other (vs. no compl. feeding)	0.016	-0.279; 0.31	-0.159	-0.369; 0.051	-0.026	-0.190; 0.138
*Lifestyle*						
Sleep at 4 months (hours)	0.009	-0.005; 0.024	**-0.011**	**-0.020; -0.002**	-0.003	-0.008; 0.002
Family screen time at 6 months (hours)	-	-	-0.008	-0.036; 0.021	-0.003	-0.014; 0.007
Possibility of unrestricted moving at 9 months (hours)	-	-	-0.007	-0.018; 0.004	-0.003	-0.009; 0.003
*Socioeconomic factors*						
Multiparity	**-0.134**	**-0.210; -0.058**	**-0.099**	**-0.156; -0.041**	-0.013	-0.036; 0.011
Maternal educational level (university vs. middle/low)	0.026	-0.057; 0.109	-0.013	-0.072; 0.045	**0.033**	**0.009; 0.056**
Household income						
€850–1150 per month (vs. < €850 per month)	0.137	-0.437; 0.711	-0.030	-0.333; 0.273	-0.096	-0.476; 0.285
€1151–3050 per month (vs. < €850 per month)	0.024	-0.497; 0.544	-0.023	-0.291; 0.245	-0.067	-0.353; 0.219
€3051–3500 per month (vs. < €850 per month)	-0.111	-0.620; 0.398	0.021	-0.271; 0.312	-0.075	-0.391; 0.241
≥ €3501 (vs. < €850 per month)	-0.037	-0.601; 0.528	-0.019	-0.334; 0.297	-0.062	-0.358; 0.235
One-parent family / co-parenthood / other (vs. two parents)	-0.033	-0.260; 0.193	0.038	-0.256; 0.331	-0.001	-0.077; 0.075
Childcare by family or friends at 3 months	0.017	-0.075; 0.109	0.013	-0.047; 0.074	-0.010	-0.034; 0.014
Mother working at 3 months after delivery	-0.055	-0.131; 0.022	0.026	-0.035; 0.087	-0.011	-0.040; 0.019

Z-score change is the change in Z-score over time, this indicates how much infants deviate from their growth curve. Explained variances of the models are shown in Table 3.

*For readability of the changes in Z-scores, all values have been multiplied by 100.

**Table 5:** Multivariate regression: factors associated with weight-for-length Z-scores change*.

	1–6 months		6–12 months		12–24 months	
*Prenatal factors*	β	95% CI	β	95% CI	β	95% CI
Gestational age (weeks)	0.032	-0.012; 0.075	-0.009	-0.037; 0.020	-0.002	-0.015; 0.010
Birth weight (kg)	**-0.372**	**-0.486; -0.258**	**0.242**	**0.155; 0.330**	**0.048**	**0.012; 0.084**
Female gender	0.089	-0.008; 0.185	-0.033	-0.101; 0.034	**-0.041**	**-0.082; -0.001**
Paternal BMI	-0.002	-0.019; 0.016	0.010	0.000; 0.020	0.001	-0.003; 0.005
Maternal pre-pregnancy BMI	0.008	-0.003; 0.020	-0.004	-0.013; 0.004	0.004	-0.003; 0.011
Gestational weight gain mother (kg)	0.004	-0.007; 0.016	-0.007	-0.016; 0.002	0.002	0.000; 0.005
Smoking during pregnancy	-0.056	-0.227; 0.114	0.003	-0.089; 0.095	0.015	-0.042; 0.073
Maternal age at date of birth	-0.005	-0.020; 0.010	0.006	-0.004; 0.016	0.001	-0.003; 0.004
Maternal diabetes	-0.218	-0.538; 0.101	-0.045	-0.222; 0.131	0.001	-0.071; 0.072
Maternal hypertension	-0.021	-0.184; 0.142	-0.032	-0.133; 0.069	0.000	-0.091; 0.091
Dutch ethnicity	0.177	-0.040; 0.394	-0.163	-0.335; 0.009	-0.005	-0.090; 0.080
*Nutrition*						
Type of feeding at 3 months						
Combined breast and bottle feeding (vs. exclusive breastfeeding)	0.066	-0.156; 0.289	**0.171**	**0.038; 0.304**	-0.039	-0.086; 0.008
Exclusive bottle feeding (vs. exclusive breastfeeding)	**0.416**	**0.284; 0.548**	0.052	-0.059; 0.162	**-0.060**	**-0.093; -0.027**
Complementary feeding at 4 months						
Fruit and/or vegetables (vs. no compl. feeding)	-0.025	-0.189; 0.138	-0.051	-0.147; 0.044	0.006	-0.037; 0.049
Porridge, possibly with fruit/veg/other (vs. no compl. feeding)	0.092	-0.085; 0.269	-0.024	-0.138; 0.089	-0.001	-0.040; 0.038
Other (vs. no compl. feeding)	0.068	-0.346; 0.482	**-0.259**	**-0.511; -0.008**	-0.082	-0.284; 0.120
*Lifestyle*						
Sleep at 4 months (hours)	0.007	-0.011; 0.025	-0.011	-0.024; 0.003	**-0.006**	**-0.012; -0.001**
Family screen time at 6 months (hours)	-	-	-0.001	-0.040; 0.039	0.000	-0.017; 0.016
Possibility of unrestricted moving at 9 months (hours)	-	-	-0.007	-0.022; 0.007	-0.006	-0.013; 0.001
*Socioeconomic factors*						
Multiparity	**-0.138**	**-0.251; -0.025**	**-0.080**	**-0.156; -0.005**	-0.011	-0.040; 0.017
Maternal educational level (university vs. middle/low)	0.107	-0.012; 0.225	-0.018	-0.096; 0.061	0.034	0.000; 0.069
Household income						
€850–1150 per month (vs. < €850 per month)	0.330	-0.439; 1.099	-0.343	-0.720; 0.034	-0.124	-0.596; 0.349
€1151–3050 per month (vs. < €850 per month)	0.197	-0.534; 0.928	-0.274	-0.574; 0.026	-0.081	-0.469; 0.306
€3051–3500 per month (vs. < €850 per month)	-0.005	-0.736; 0.727	-0.200	-0.539; 0.138	-0.071	-0.502; 0.360
≥ €3501 (vs. < €850 per month)	0.172	-0.605; 0.949	-0.191	-0.556; 0.175	-0.099	-0.496; 0.299
One-parent family / co-parenthood / other (vs. two parents)	-0.102	-0.400; 0.197	0.012	-0.353; 0.376	0.000	-0.100; 0.101
Childcare by family or friends at 3 months	0.001	-0.152; 0.153	-0.005	-0.078; 0.068	-0.001	-0.029; 0.026
Mother working at 3 months after delivery	-0.026	-0.137; 0.089	0.065	-0.024; 0.155	-0.022	-0.052; 0.008

Z-score change is the change in Z-score over time, this indicates how much infants deviate from their growth curve. Explained variances of the models are shown in Table 3.

*For readability of the changes in Z-scores, all values have been multiplied by 100.

### First six months

**Fig 1:**
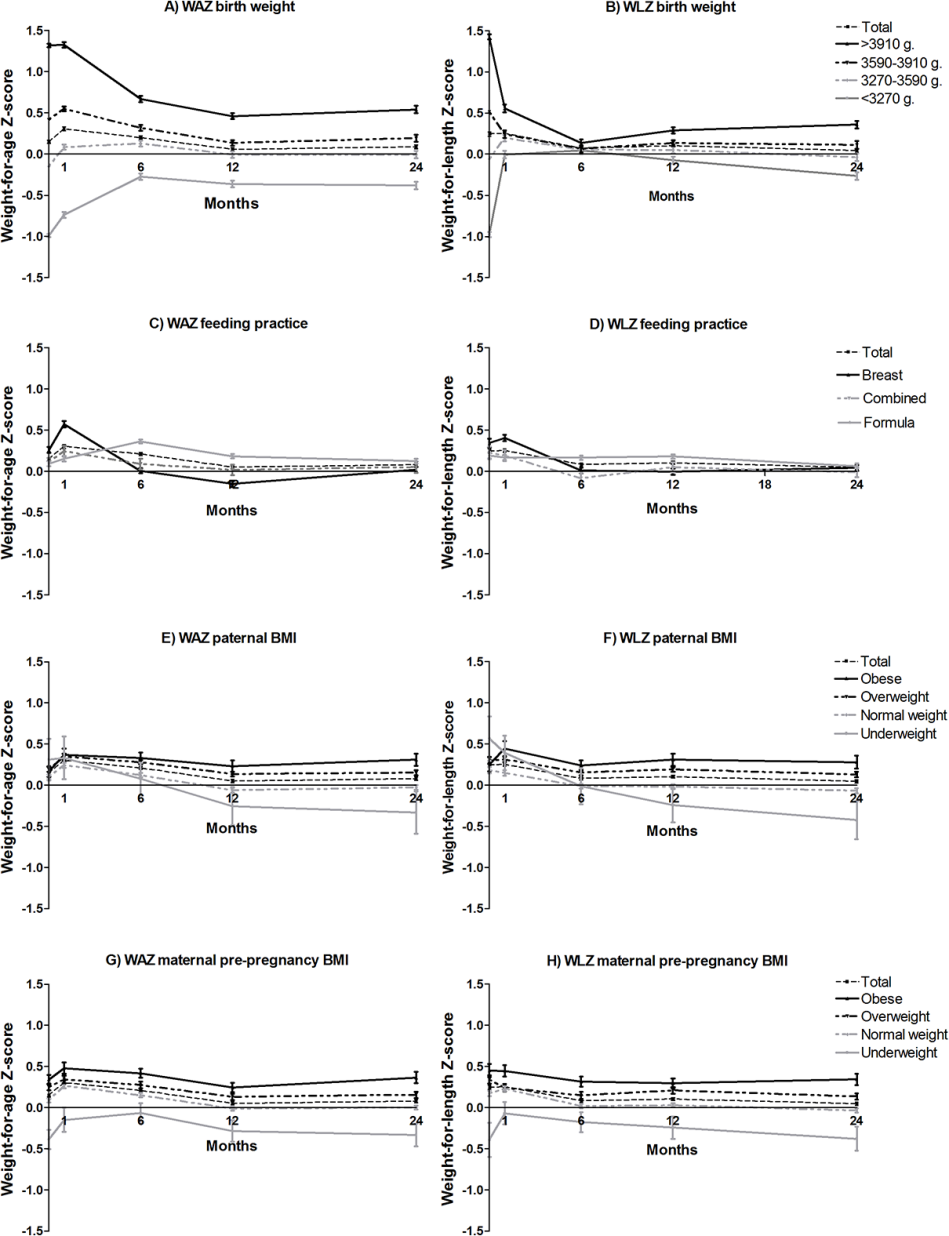
Mean weight-for-age and weight-for-length Z-scores, stratified for birth weight (A-B), type of feeding (C-D), paternal BMI (E-F) and maternal pre-pregnancy BMI (G-H).

**Fig 2:**
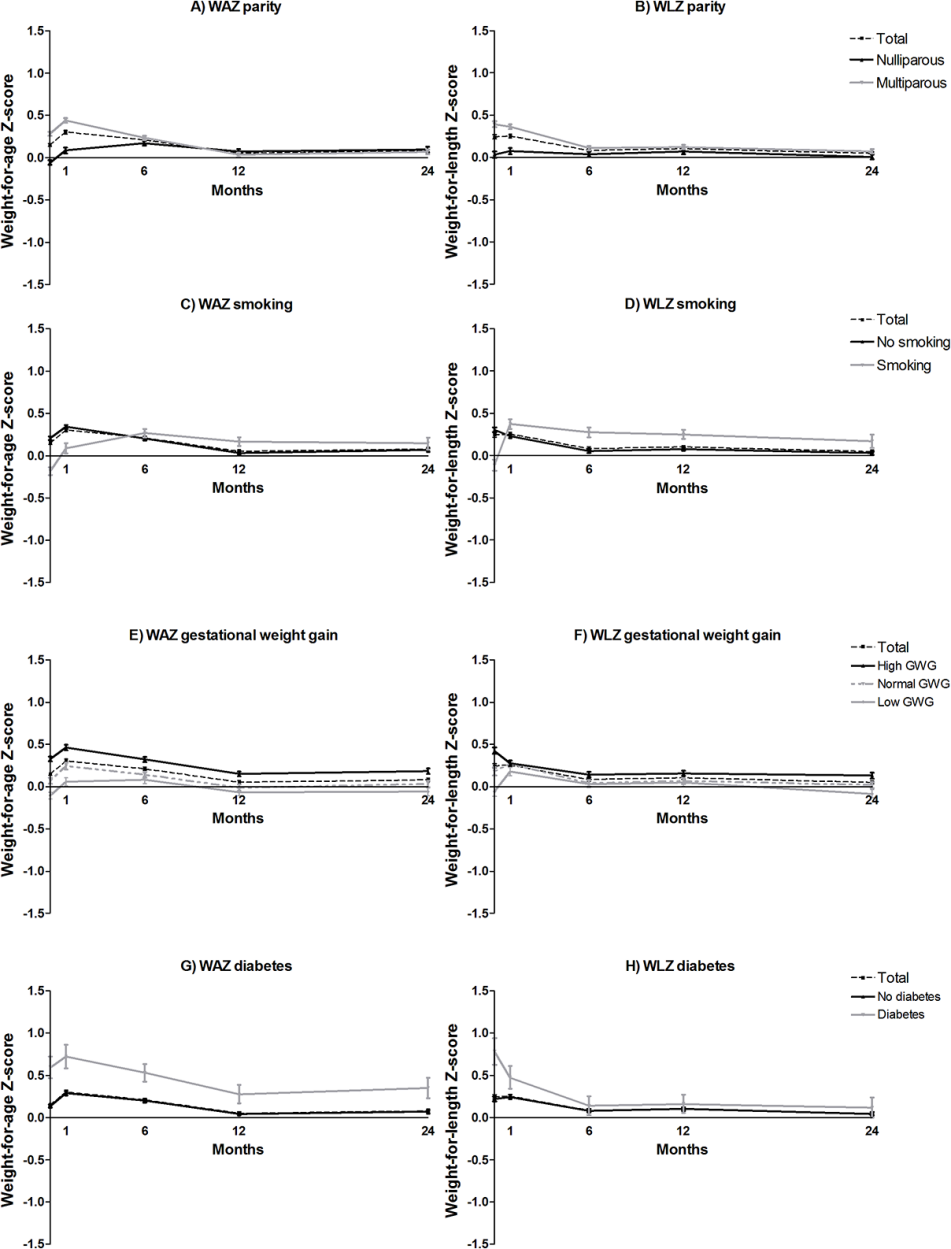
Mean weight-for-age and weight-for-length Z-scores, stratified for parity (A-B), smoking (C-D), gestational weight gain (E-F) and diabetes (pre-existing and gestational) (G-H).

From one to six months, a higher gestational age, higher birth weight and female gender were all associated with a decline in weight-for-age Z-score (Table 4). Furthermore, a higher birth weight was associated with a decline in weight-for-length Z-score (Table 5). For hypothesis-generating purposes and to facilitate the interpretation of the results, the crude, unadjusted patterns for the Z-scores over time of all variables are shown in Figs 1 and 2 and S1–S4 Figs. Breastfed infants showed a rapid increase in weight-for-age and weight-for-length Z-score in the first month of life and a decrease between 1–6 months, while formula fed infants showed a continuous increase in weight-for-age Z-score during the first six months (Fig 1). At six months, formula fed infants had a higher weight-for-age Z-score and weight-for-length Z-score compared to breastfed infants (Tables 4 and 5, Fig 1C and 1D). In the lowest birth weight quartile we observed a similar growth pattern: a quick weight gain for breast fed infants and a slower weight gain for formula fed infants (data not shown). In this period, multiparity was also associated with a decline in weight-for-age Z-score and weight-for-length Z-score (Tables 4 and 5, Fig 2A and 2B). From one to six months the prenatal domain was most important to explain differences in weight gain. It explained 26.6% of the changes in weight-for-age Z-score and 3.7% for weight-for-length Z-score (Table 3). This was followed by the nutrition domain, which explained 10.7% of variation in weight-for-age Z-score and 2.8% for weight-for-length Z-score changes over time.

### Six to twelve months

From 6–12 months, higher birth weight, higher paternal BMI, more hours of sleep and multiparity were associated with declines in weight-for-age Z-scores. Furthermore, higher birth weight and combined breast and formula feeding were associated with increased weight-for-length Z-scores. Complementary feeding and multiparity were associated with decreased weight-for-length Z-scores (Tables 4 and 5, Fig 2A and 2B. and S4A and S4B Fig). The percentage of variation in Z-scores that could be explained decreased considerably after 6 months of age (Table 3). Still, the prenatal domain was most important to explain weight gain between 6–12 months (R^2^ = 1.8% for weight-for-age and 3.8% for weight-for-length).

### Twelve to twenty-four months

A higher birth weight was associated with an inclined weight-for-length Z-score (Tables 4 and 5). Furthermore, after the incline between 1–6 months, we observed a decline in weight-for-age and weight-for-length Z-score for formula fed infants compared to breastfed infants (see also Fig 1C and 1D). More hours of sleep was associated with a decline in weight-for-length Z-score. Higher maternal education was associated with increased weight-for-age Z-score (see also S1E and S1F Fig). Between 12 and 24 months we could still explain very little of the variation in growth velocities, but the nutritional domain was most important for weight-for-age Z-score (3.1%) and the prenatal domain for weight-for-length Z-score (2.6%), see Table 3.

### Comparing periods

Overall, with all predictors together we could explain 38.7% of the variation in weight-for-age Z-scores in the first six months, which decreased to 4.5% in the second six months and 6.9% for the second year (Table 3). Furthermore, we could explain 7.5%, 6.3% and 5.8% of variation in weight-for-length Z-scores in these three periods respectively (Table 4). Opposing effects in different periods are illustrated in Figs 1 and 2, e.g. a decline in Z-score for breastfed infants in the first six months versus an incline for these same infants in the second year of life. We did not observe a clear association between maternal pre-pregnancy BMI and weight gain in the first two years. However, Fig 1G and 1H show that obese mothers had heavier babies, and these higher absolute Z-scores remained consistent over the first two years of life. Several other factors showed a similar influence (e.g. maternal diabetes, gestational weight gain and complementary feeding).

## Discussion

A number of studies identified multiple factors that explain infant growth [13,15,18,19]. Apart from identifying the individual effects, the question which domains, and within the domains which specific factors are dominant is not answered yet. In this study we observed that the prenatal and nutritional domain were most importantly associated with infant growth in terms of weight-for-age and weight-for-length. Within these domains, birth weight and type of feeding were best at explaining differences in growth patterns.

The association between low birth weight and positive infant weight gain had been described before [11,12,26]. It is an important observation in the catch-up growth hypothesis which suggests that rapid catch-up may lead to overweight later in life [7,8,11]. However, the association between birth weight and weight gain is not a simple matter of “the lower the birth weight, the more rapid the growth”. We found that birth weight had the largest impact on weight gain in the first six months of life, after which its influence drastically reduced. All infants showed growth towards the group mean in the first six months, with high birth weight infants directing towards a lower weight-for-age Z-score and lower birth weight infants directing towards a higher weight-for-age Z-score. A similar convergence of growth patterns of infants with different birth weights was also reported by Ong et al. [13]. Apparently, there is a strong drive to compensate for antenatal restraint or enhancement of fetal growth caused by intrauterine factors. Interestingly however, although the effect of birth weight on gain in weight-for-age seemed to stabilize over time, its effect on gain in weight-for-length persisted. This resulted in an inverse overall association between birth weight and weight-for-length in the first six months, with a positive association later in life, between 6 and 12 months, and 12 and 24 months. This could indicate fetal programming, in which prenatal factors influence very early development [27].

The second most important domain was nutrition, in particular the type of feeding. The pattern of weight gain differed for breast and formula fed infants. In some periods breastfed infants grew faster, while in other periods formula fed infants grew faster. The evidence for a lower growth rate in breastfed children compared to formula fed children has been rather consistent [11,28–30]. Our results indicated that this did not hold for the whole first year of life. We observed that breastfed infants showed a rapid weight gain directly after birth with no change in Z-scores from one month onwards, whereas formula fed infants gained weight slower, peaked at 6 months of age and remained to have a higher Z-score thereafter. Druet et al. [31] showed a higher weight in formula fed infants after six months. Their group previously showed that both breast and formula fed infants showed a growth towards the mean [13]. However, they showed no data on growth patterns in the first six months of life. In the lowest birth weight quartile, which was mainly in the normal range (1630g–3270g, of which 5% <2500g), we observed a similar growth pattern: a quick weight gain for breast fed infants and a slower weight gain for formula fed infants. Thus, a rapid growth in these smaller babies also existed in breast fed infants. However, in this study we were interested in the growth within the normal range, thus in rapid growth instead of catch-up growth in preterm or very low birth weight infants. Therefore it could be debated whether this rapid growth is a factor causing obesity later in life, because smaller babies showed a growth towards the mean and not an excessive growth beyond the mean, in both breastfed and formula fed infants. The rapid weight gain during the first month of life in breastfed infants might be related to a high intake of breast milk during that period. Whitehead showed already 20 years ago, using the doubly labelled water method, that the breast milk intake rapidly increased after birth with hardly any change in the period of two to four months of life [32]. Whitehead showed that the total caloric intake of a two month old breastfed infant hardly differed from a breastfed infant of 4 month. This differed from formula fed infants, where the advised intake is related to weight. These caloric intake patterns fitted the different weight gain patterns for breastfeeding vs formula feeding in our study very well. Whether the long-term differences in weight gain patterns (e.g. during adolescence) are related to this difference in weight gain pattern directly after birth is unclear. It also raises the question if the difference in risk to develop overweight between breast and formula fed infants is primarily related to the quality and composition of milk or to this difference in caloric intake pattern. Furthermore, the “protective effect” of breast feeding might be the result of a healthier lifestyle overall [13].

Other factors that significantly explained differences in weight gain between children were gestational age, gender, and paternal BMI from the prenatal domain, complementary feeding from the nutrition domain, hours of sleep at 4 months of age from the lifestyle domain, and parity and maternal educational level from the socioeconomic domain. These factors have previously also been shown to be associated with childhood obesity [11,13,14,19]. Maternal smoking did not show a significant association with growth, however, a trend towards a lower birth weight for infants of smoking mothers was found, followed by an increased weight-for-age Z-score until six months of age. After six months of age, infants of smoking mothers showed a slightly higher weight-for-age and weight-for-length Z-score compared to infants of non-smoking mothers. This rapid infant growth is in line with previous studies, although many studies showed a clear increased weight later in childhood as well [13,33,34].

The analyses in this paper focussed on the most important determinants for weight-for-age and weight-for-length growth velocity. However, we also identified some interesting factors that were not significantly related to the rate of weight gain, but had a consistent effect on Z-score over time, without a change in actual Z-score. An example is a high maternal pre-pregnancy BMI which was associated with a higher birth weight and with a consistently higher weight-for-age and weight-for-length Z-score [35–38]. Since body weight is subject to tracking, this can also be an important determinant for body weight later in life [22].

With regard to mutual comparison of the relative influence of potential risk factors in different time periods in the first years of life, little is known from literature. Many papers have analysed data over a larger growth periods, e.g. 0 to 12 months [13] or longer [14,15]. In the current study, the importance of birth weight and type of feeding for infant growth was mainly found for the first 6 months of life. This means that although it seemed that birth weight for example determined growth in the first two years of life rather weakly, it was in fact strongly influencing the first six months of life. Indeed, the main association of birth weight seemed rather small and inverse over the total period of 1 to 24 months (data not shown). This was the result of a combination of a strong inverse association over 1–6 months and smaller or no association over 6–24 months; a larger time frame caused a dilution of the associations. The selected factors in our study only explained a minor part of the weight gain after six months of age. Another issue was the direction of effect. The overall effect of birth weight on weight gain in the first six months seemed rather weakly inverse. However, in reality it was a combination of a strong positive association, i.e. an increased Z-score for low birth weight infants, and a strong negative association, i.e. a decreased Z-score for high birth weight infants. Thus, in such a dynamic period as the first two years of life, it is important to avoid oversimplification and to distinguish subgroups and the right time frame to correctly interpret the results.

Apart from this methodological issue, separating time periods may also be important for prediction. Interest in the determinants of infant weight gain is partly inspired by the drive to tackle the childhood obesity epidemic. Two categories of risk factors seemed to exist. First, those that explained the baseline risk for future overweight and had a permanent and consistent effect on weight-for-length Z-scores, e.g. maternal pre-pregnancy BMI. Second, those that were related to growth velocity, driving Z-scores to higher growth curves, e.g. paternal BMI. This difference in risk factors had been suggested before [14]. Furthermore, although birth weight affected changes in weight-for-length Z-score after the first six months, the explained variation was very low. At the same time, factors like paternal BMI became more important. This suggests that other, yet undetermined, factors like genetics or shared lifestyle or environment become more important over time [39][36,38].

Although their means and standard errors were comparable, six factors showed minor differences in the significance of their association with change in weight-for-age Z-scores between the imputed and the original dataset: paternal BMI (original data 6–12mo: β = 0.006 [95%CI = -0,008;0,02]), maternal BMI (original data 12–24mo: β = 0.007 [95%CI = 0.002;0.012]), smoking (original data 12–24mo: β = 0.081 [95%CI = 0.021;0.142], maternal age (original data 6–12mo: β = 0.014 [95%CI = 0.002;0.027], unrestricted moving (original data 12–24mo: β = -0.013 [95%CI = -0.023;-0.003] and maternal education (original data 1–6mo: β = 0.128 [95%CI = 0.001;0.255]. Meta-analysis is necessary to confirm our results on these factors.

### Strengths and limitations

A strength of this study was the prospective investigation of change in weight-for-age and weight-for-length in different periods in the first two years of life, using repeated measurements, comparing three different age periods, and the large sample size. Another strength was the hypothesis driven approach to select relevant variables [16]. All variables were selected based on a priori association with infant growth and weight changes. Despite the large number of variables that were investigated, the hypothesis-based approach reduced the risk of type I error (incorrect rejection of the null hypothesis). Our aim was set up in a two-step approach: 1) to compare the explained variance of growth by individual predictors and 2) to compare these explained variances of growth within the four domains. These domains were based on the theoretical health fields framework of Lalonde: human biology (prenatal factors), environment (socioeconomic factors) and lifestyle (nutrition and lifestyle) [16]. Additionally, the inclusion of sleep, screen time and unrestricted moving, which were not very widely studied in this age group before, added value to this study [40]. Another strength was the inclusion of paternal determinants for prediction of growth, because these paternal factors are often not included. We did not use a polynomial function for longitudinal growth, which could be seen as a limitation. However, such a function could “overfit” the model, which could cause peaks in growth curves to be exaggerated [13]. Furthermore, the decision to analyse time frames separately allowed comparing the specific growth periods, which would not be possible for longitudinal growth curves. A limitation for clinical relevance could be that the some effect sizes seemed rather small. This could be due to the fact that results were expressed as changes in Z-score, however a change of >0.5 Z-score could be relevant at the population level. Furthermore, the effects for individuals differing from the mean were likely to be clinically relevant, as shown in the figures. For example, within the low birth weight group, the mean weight-for-age Z-score at 1 month was -0.74 SD. If infants grew following their expected growth curve (Z-score change = 0.000), their Z-score at 6 months would still be -0.74, which corresponds to an absolute weight of 6684 grams. However, the observed weight-for-age Z-score at 6 months was -0.27, which corresponds to an absolute weight of 7107 grams. This is a difference of 423 grams (6%) in body weight.

### Conclusion and perspectives

We found that the prenatal and the nutrition domain were most important to explain the variation in weight gain in the first two years of life. This variation could best be explained in the first half year of life. After six months of life other yet unknown factors start to play a role.

## Supporting Information

S1 Fig Mean weight-for-age and weight-for-length Z-scores, stratified for gender (A-B), hours of sleep (C-D), maternal education (E-F), maternal age (G-H). (TIF)

S2 Fig Mean weight-for-age and weight-for-length Z-scores, stratified for gestational age (A-B), ethnicity (C-D), hypertension (E-F) and family structure (G-H). (TIF)

S3 Fig Mean weight-for-age and weight-for-length Z-scores, stratified for family screen time (A-B), hours of unrestricted moving (C-D), childcare (E-F) and mother started working at 3 months (G-H). (TIF)

S4 Fig Mean weight-for-age and weight-for-length Z-scores, stratified for complementary feeding (A-B) and household income (C-D). (TIF)
